# The Effects of Objective and Subjective Socioeconomic Status on Subjective Well-Being among Rural-to-Urban Migrants in China: The Moderating Role of Subjective Social Mobility

**DOI:** 10.3389/fpsyg.2017.00819

**Published:** 2017-05-22

**Authors:** Silin Huang, Jiawei Hou, Ling Sun, Donghui Dou, Xia Liu, Hongchuan Zhang

**Affiliations:** ^1^Institute of Developmental Psychology, School of Psychology, Beijing Normal UniversityBeijing, China; ^2^School of Sociology and Psychology, Central University of Finance and EconomicsBeijing, China

**Keywords:** objective socioeconomic status, subjective socioeconomic status, subjective well-being, subjective social mobility, Chinese rural-to-urban migrants

## Abstract

Although previous investigations have agreed that Chinese rural-to-urban migrants’ socioeconomic status (SES) increases with their migration, the association between SES and subjective well-being is uncertain. To address this research gap, the present study proposed that the association between objective SES and subjective well-being is mediated by subjective SES. This model was tested with a sample of 432 Chinese rural-to-urban migrants. The results indicate a significant association between objective SES and subjective well-being and a partial mediating effect of subjective SES. Furthermore, subjective social mobility, which is one’s expectation about the possibility to move upward in the social hierarchy, was found to moderate both the direct path from objective SES to subjective well-being and the indirect path from subjective SES to subjective well-being. These findings suggest that Chinese rural-to-urban migrants gained in subjective well-being not only because of direct financial achievement but also because of their perceptions and beliefs about their relative social status.

## Introduction

Millions of Chinese rural-to-urban migrants have played an important role in maintaining China as the world’s fastest-growing major economy and leading the world to economic recovery following the recent global recession (Ramzy, 2009). The migrants referred to in the present study are those who move from rural areas to towns and cities seeking better living conditions without possessing a permanent urban household registration in China (*HuKou*) (China National Bureau of Statistics [CNBS], 2015). The number of Chinese rural-to-urban migrants is already the greatest in human history (Knight and Gunatilaka, 2010) and will continue to expand rapidly in future decades. According to the latest population statistics bulletin, there were 273.95 million migrants in 2014, an increase of 5.01 million over the previous year (China National Bureau of Statistics [CNBS], 2015). In Beijing, more than 70% of the impermanent populations of 8.18 million people were rural-to-urban migrants in 2014 (Beijing Municipal Bureau of Statistics [BMBS], 2015). To raise their socioeconomic status (SES), the Chinese government’s recent policy of new-type urbanization further encouraged more migrants to settle in urban areas with their families.

A number of empirical investigations have shown that migrants’ SES has increased with migration in recent decades. Based on the national investigation bulletin in 2014, almost all the observable measures of migrants’ SES, including income, employment, living conditions, and welfare, have improved significantly in the past decades (China National Bureau of Statistics [CNBS], 2015). Lu (2012) analyzed the long-run wage tendencies of migrants and found that it grew at an annual rate of 10% from 1979 to 2010. Knight and Gunatilaka (2010) showed that the average income per capita of migrant families is 2.9 times of that of rural families. Similar results were also found in other studies (Li, 2007; Zhu, 2010). Taken together, mounting evidence has demonstrated that Chinese rural-to-urban migrants have indeed achieved higher SES by moving from rural areas to urban areas.

Despite the high attention paid to the observable socio-economic achievements described above, whether attaining higher SES leads to an increased feeling of well-being is a far more important social issue (McGillivray, 2007; Van Hoorn, 2008; Knight and Gunatilaka, 2010; Chen, 2013). Migration theory posits that the main reasons for migration are better jobs and higher income, which further provide a means to maximize happiness (Knight and Gunatilaka, 2010; Den Berg, 2011). Millions of migrants have moved out of rural areas not only to escape poverty and misery but also to seek healthier and happier lives. Improving the well-being of migrants is also a fundamental goal of new-type urbanization in present China. Generally, the feeling of well-being has been measured using so-called subjective well-being, comprising cognitive judgments of life satisfaction and affective evaluations of emotions and moods (Diener et al., 1999, 2002). However, previous studies still have not reached a consistent conclusion about the association between SES and subjective well-being in Chinese rural-to-urban migrants. Some studies found a strong correlation between the two variables. For instance, Knight and Gunatilaka (2010) found that lower income was the predominant reason for migrants’ unhappiness. Li (2007) suggested that compared with their past experience living in rural areas, 55.9% of migrants reported that they enjoyed happier lives with improved SES in their current urban location. Similarly, Lin (2007) showed that 68.1% of migrants reported a stronger sense of happiness in their sample. This conclusion has been supported by other investigations (Zhang, 2007). In support of this line of findings, Howell and Howell (2008) analyzed 111 independent samples from 54 economically developing countries and found that the effect size of the relationship between SES and subjective well-being is stronger for those with a lower income and education level. Undoubtedly, Chinese rural-to-urban migrants make up the majority of the people with lower income and education levels in the social hierarchy. Therefore, previous findings seem to suggest that SES effectively predicts Chinese rural-to-urban migrants’ subjective well-being.

However, increasing evidence has suggested that SES has only a negligible impact on migrants’ subjective well-being. Recent studies found that migrants’ subjective well-being did not differ significantly from that of rural residents (Chen, 2013) or was even lower than that of rural peasants and urban citizens (Knight and Gunatilaka, 2010). A latest research (Easterlin et al., 2012) also revealed that despite the rapid economic growth of the past two decades, China’s lower-income group’s life satisfaction decreased from 1990 to 2007. This finding is in line with the increasing evidence from different samples suggesting that higher SES contributes little to one’s happiness (Diener et al., 1999; Boyce et al., 2010; Anderson et al., 2012). Altogether, these findings seem to indicate that the association between SES and subjective well-being among Chinese rural-to-urban migrants is weaker than expected. In another words, SES does not always bring happiness to Chinese rural-to-urban migrants.

Therefore, there may exist variables that mediate or moderate the effect of SES on migrants’ subjective well-being. Here, we present a potential explanation for the contradictory findings: that previous studies focused mainly on the observable measurements of SES, or so-called objective SES (Clark and Oswald, 1994; Adler et al., 2000; Nettle, 2005; Kraus and Stephens, 2012), thus neglecting the subjective SES, which is defined as an individual’s perception of where he/she resides on the social ladder (Adler et al., 2000; Demakakos et al., 2008; Anderson et al., 2012; Kraus and Stephens, 2012; Kraus et al., 2013a; Curhan et al., 2014).

Objective SES is the economic and social position in relation to others, which is widely measured by using three indicators: income, education, and occupation. In contrast, subjective SES is a person’s conception of his or her position compared with that of others (Anderson et al., 2012; Kraus and Stephens, 2012). Although objective SES provides the material basis for subjective SES (Demakakos et al., 2008), the correlation between the two variables is only moderate. For instance, Adler et al. (2000) found that the correlations between subjective SES and the three indicators of objective SES ranged from 0.11 to 0.32. Similar results were reported in other investigations (Ostrove et al., 2000; Gong et al., 2012). Moreover, the extent of the correlation coefficients varied in different samples. Ostrove et al. (2000) discovered that subjective SES was significantly related to educational attainment, household income, and occupation in White, Latina, and Chinese Americans but not in African Americans. Therefore, subjective SES may work beyond objective SES to influence its psychological effects.

In support of this line of findings, an increasing number of investigations have found that compared with objective SES, subjective SES exhibits a more stable and intense association with psychological functioning, such as negative affectivity, stress, and pessimism (Demakakos et al., 2008; Lei and Tam, 2012; Kraus et al., 2013b), health-related factors (Adler et al., 2000; Demakakos et al., 2008; Gong et al., 2012; Lei and Tam, 2012; Kraus et al., 2013a; Quon and McGrath, 2014), cognition, empathy, and behavior (Kraus et al., 2010, Kraus et al., 2011). Hence, we hypothesize that due to the more proximal association between subjective SES and subjective well-being, it may mediate the effect of objective SES on subjective well-being among Chinese rural-to-urban migrants. We based our hypothesis on several related studies. First, subjective SES is an immediate measure that can discriminate subtle but substantial variations in the social environment that objective SES cannot access. For example, subjective SES may differ within jobs belonging to the same occupation but with dissimilar types of work, such as famous professors versus ordinary instructors (Gong et al., 2012). Second, one previous study found that subjective SES more strongly predicted psychological well-being than objective SES in a United States sample (Curhan et al., 2014). Additionally, research exploring the impact of SES on health revealed that subjective SES mediates fully or partially the associations between objective SES and depression (Demakakos et al., 2008; Gong et al., 2012). Based on the above literature, we hypothesize that the correlations between objective SES, subjective SES and subjective well-being among rural-to-urban migrants in China are significant and positive (H1). However, in addition to the significant direct effect of objective SES on subjective well-being (H2a), subjective SES serves as a mediator between objective SES and subjective well-being in Chinese rural-to-urban migrants (H2b).

A further hypothesis is that subjective social mobility may serve as a moderating factor. Subjective social mobility is defined as people’s subjective belief that they have a higher probability to reach a higher social class in future, especially in comparison to their parents’ positions (Fischer, 2009; Kelley and Kelley, 2009; Den Berg, 2011; Huang et al., 2016). Different from actual social mobility, subjective social mobility focuses on one’s attitude toward inequality (Kelley and Kelley, 2009) and one’s expectations about moving up on the social ladder (Den Berg, 2011). Large-scale surveys on subjective social mobility support this moderating hypothesis. For example, Fischer (2009) found that subjective social mobility mitigates the negative effect of income inequality on people’s subjective well-being and that living in a socially mobile society is beneficial to individual life satisfaction. Kelley and Kelley (2009) found that subjective social mobility has a notable effect on subjective social class and attitude toward inequality, even after controlling for actual social mobility, income, and class. In summary, these results suggest that subjective social mobility can help disadvantaged Chinese rural-to-urban migrants to adapt to the urban life. It is reasonable to hypothesize that subjective social mobility moderates both the direct path from objective SES to subjective well-being (H3a) and the indirect path from objective SES to subjective well-being through subjective SES, including the effects of objective SES on subjective SES (H3b) and subjective SES on subjective well-being (H3c), among Chinese rural-to-urban migrants.

## Materials and Methods

### Participants and Procedure

There were three eligibility criteria for selecting participants in the present study: (1) their ages range from 18 to 60 years old. Most migrants more than 60 years old are retirees or return to their hometown in China. This limit was set to avoid a sampling bias. (2) They work or stay in Beijing without a permanent *hukou* in the city. *Hukou* is an individual’s registration status in a certain administrative-geographical unit and is normally denoted as rural or urban. Therefore, *hukou* information can help to identify participants as real rural-to-urban migrants. (3) They had been living in Beijing for at least 3 months. A total of 432 Chinese rural-to-urban migrants with rural *hukou* aged from 19 to 60 years were recruited, and the mean age was 31.88 years (*SD* = 8.47), with 44.2% females.

The investigation was conducted in 2014 in the Haidian and Changping districts in Beijing, two districts primarily inhabited by rural-to-urban migrants. Trained interviewers met participants on streets and identified participants with the above criteria. All participants who consented to participate were requested to subsequently complete the anonymous questionnaire and received a small amount of compensation (about $5) for their participation. Interviewers read the items aloud to some illiterate participants, and the participants provided oral answers to the interviewers, who faithfully recorded the answers in questionnaires. The total process took approximately 50 min, and the whole study was completed in 2 weeks. The investigation protocol was approved by the Institutional Review Boards at Beijing Normal University and Central University of Finance and Economics in China.

### Measures

#### Demographic Form

Participants’ background information including age, gender, marital status (i.e., unmarried, married, divorced, and widowed), and *hukou* were recorded in a brief demographic form.

#### Subjective Well-Being

Participants’ subjective well-being was measured by the Satisfaction with Life Scale (SWLS) and the Positive Affect and Negative Affect Scale (PANAS). The SWLS includes five items assessing overall self-judgments of respondents’ lives (Diener et al., 1999). Participants were asked to indicate their feelings on a 7-point scale ranging from 1 (lowest satisfaction with life) to 7 (highest satisfaction with life). The mean of the five items was calculated and then transformed into standardized scores. Cronbach’s alpha of the SWLS was 0.78.

The PANAS includes a positive affect scale (PAS) and a negative affect scale (NAS), which surveyed whether participants frequently experienced certain emotions recently (Watson et al., 1988). Participants were asked to respond to 20 items for the PAS and the NAS with answers ranging from 1 (none of the time) to 5 (most of the time). Cronbach’s alpha of the PAS was 0.80, and that of the NAS was 0.82 individually.

As in previous research (Anderson et al., 2012), we formed an overall index of subjective well-being by standardizing scores on each of the three indicators after the NAS items were reversely coded, and the scores were then averaged. Previous investigations found that these measures are reliable and valid for Chinese rural-to-urban migrants (Du and Liu, 2011; Xing et al., 2012).

#### Objective SES

Objective SES was indexed by personal monthly income in the present study because migrants’ income is a significant consequence of migration (Li, 2007; Li and Li, 2010; Lu, 2012; China National Bureau of Statistics [CNBS], 2015). Income is the most frequently used and reliable indicator of objective SES (Johnson et al., 2011; Kraus and Stephens, 2012). Participants were asked to factually report their monthly income, which was transformed into a natural logarithm, and its standard score was used (Boyce et al., 2010; Johnson et al., 2011).

#### Subjective SES

We adopted the MacArthur Scale to assess migrants’ subjective SES (Adler et al., 2000). A diagram of a ladder with 10 rungs was shown to participants and described as follows (Adler et al., 2000): “the rungs in the ladder represent the social classes where people stand in our society. At the top of the ladder are the people who are the best off, those who have the most income, most education, and best jobs. At the bottom are the people who are the worst off, those who have the lowest income, least education, and worst jobs or no job.” Participants were asked to make a comparison to their acquaintances (e.g., friends, family, and work group) around themselves and marked the rung that best represents where they believe they stand on the ladder. Participants’ scores ranged from 1 (bottom rung) to 10 (top rung) and were transformed to standard scores. The measure was used previously for Chinese rural-to-urban migrants and has shown to be reliable and valid (Jin, 2016).

#### Subjective Social Mobility

The six-item subjective social mobility scale (Ming, 2013) was used to assess participants’ level of subjective social mobility. These items were as follows: “*According to the present society, I am capable of improving my social status.*” “*Compared with my parents, my social status will be higher in the future.*” “*Compared with my companions, I have a higher social status now.*” “*I will achieve a higher social status in the future.*” “*I could improve my social situation provided I strived constantly.*” “*My social class will be higher than my companions’ in the future.*” Participants rated how well each item fit with their experience on a scale from 1 (strongly disagree) to 5 (strongly agree). The means of all six items were computed and then transformed into standard scores. This scale has been used among Chinese undergraduates with adequate reliability (Ming, 2013; Huang et al., 2016). Cronbach’s alpha of the present sample was 0.80.

### Data Analyses

Previous literature has suggested that gender, marital status, and age may be effective predictors of subjective well-being (Diener et al., 1999; Zhang, 2007; Chen, 2013); hence, these three factors were controlled for in the following analysis. The two nominal variables (gender and marriage) were dummy-coded individually (0 = female, 1 = male; 0 = unmarried, 1 = married), and other continuous variables were standardized.

The first analysis was conducted to test the associations between objective SES, subjective SES, subjective social mobility, and subjective well-being among migrants. The second analysis examined the model that subjective SES mediates the effect of objective SES on subjective well-being. The third analysis tested the models that subjective social mobility moderates the direct effect of objective SES on subjective well-being and the indirect effect of objective SES on subjective well-being through subjective SES.

We used IBM SPSS version 19 to perform the correlation analyses. For the latter two analyses, we used the PROCESS macro^1^ (Hayes, 2013, 2016) to perform bias-corrected bootstrapping multiple-mediation analyses with 5000 resamples. Multiple-mediation analyses have the benefit of examining the indirect effects through subjective SES and the moderating role of subjective social mobility while controlling for the effects of the other factors simultaneously. The mediating effects of subjective SES between objective SES and subjective well-being were tested by using Model 4. The moderating roles of subjective social mobility on the above effects were tested using Model 59.

## Results

### Descriptive Statistics and Correlations between the Main Variables

**Table 1** shows the means, standard deviations, and zero-order correlations between the main variables’ measures in this study. Consistent with H1, there were significant and positive correlations between objective SES, subjective SES and subjective well-being. Additionally, subjective social mobility was significantly positively correlated with objective and subjective SES and subjective well-being separately. To control for a potential Type I error accumulation, we used the Benjamini–Hochberg procedure to correct the level of statistical significance of correlation coefficients (Benjamini and Hochberg, 1995). The false-discovery rate (FDR) of 5% was applied to adjust all *p*-values resulting from correlation coefficients in **Table 1**. The results suggested that all correlations remained statistically significant after Type I error accumulation was controlled.

**Table 1:** Descriptive statistics and zero-order correlations of main variables (*N* = 432).

Variables	*M*	*SD*	1	2	3
(1) Objective socioeconomic status	8.25	0.65	–	–	
(2) Subjective socioeconomic status	4.94	2.36	0.12^∗^	–	–
(3) Subjective social mobility	3.46	0.86	0.10^∗^	0.22^∗∗∗^	–
(4) Subjective well-being	0.00	2.03	0.14^∗∗^	0.15^∗∗∗^	0.33^∗∗∗^


*^∗^*p* < 0.05, ^∗∗^*p* < 0.01, ^∗∗∗^*p* < 0.001. (All correlations were still statistically significant when the false-discovery rate at 5% was controlled for).*

### The Mediating Effect of Subjective SES on the Association between Objective SES and Subjective Well-Being

**FIGURE 1:**
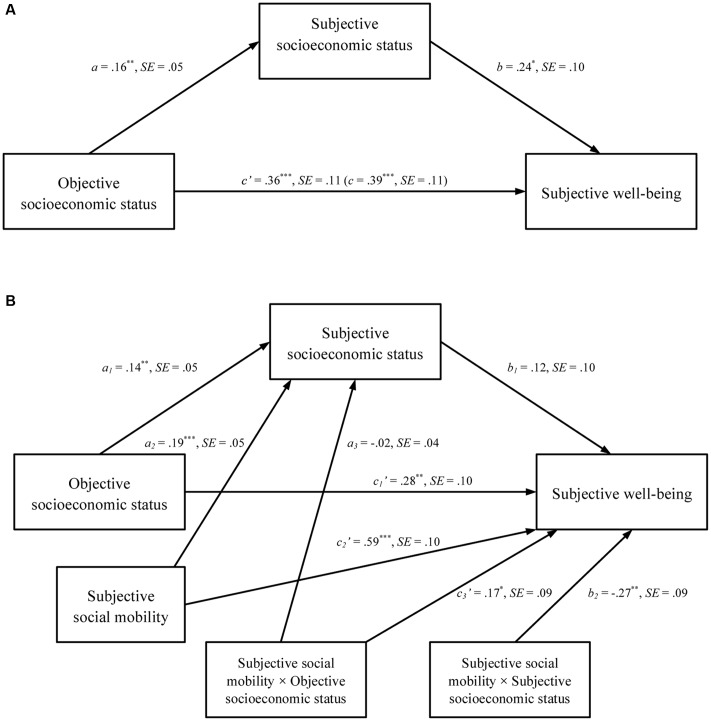
**Mediation showing unstandardized coefficients for the direct and indirect paths from objective socioeconomic status to subjective well-being among Chinese rural-to-urban migrants** **(A)** through subjective socioeconomic status and **(B)** moderated by subjective social mobility. Asterisks indicate statistically significant coefficients (^∗^*p* < 0.05).

We found that the mediation model was significant; γ = 0.04, *SE* = 0.02, 95% CIs = [0.01, 0.10], *R^2^_Model_* = 0.08, Δ*R*^2^*_Addition of Mediators_* = 0.02. The results of the path in the mediation are shown in **Figure 1A**. Consistent with the mediation hypothesis H2b, we found a significant indirect path from objective SES to subjective well-being through subjective SES. In line with hypothesis H2a, the direct path between objective SES and subjective well-being was still significant after subjective SES was entered into the analysis, indicating that the relationship between objective SES and subjective well-being is only partially mediated by subjective SES. The mediation effect size that describes the ratio of the indirect effect to the total effect (ab/c) was 0.10.

### The Moderating Role of Subjective Social Mobility on the Effects of Objective and Subjective SES to Subjective Well-Being

**FIGURE 2:**
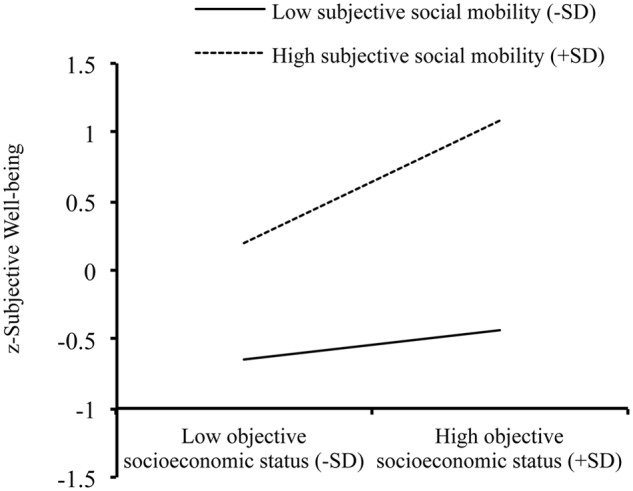
**Subjective social mobility as a moderator of the association between objective socioeconomic status and subjective well-being**.

**FIGURE 3:**
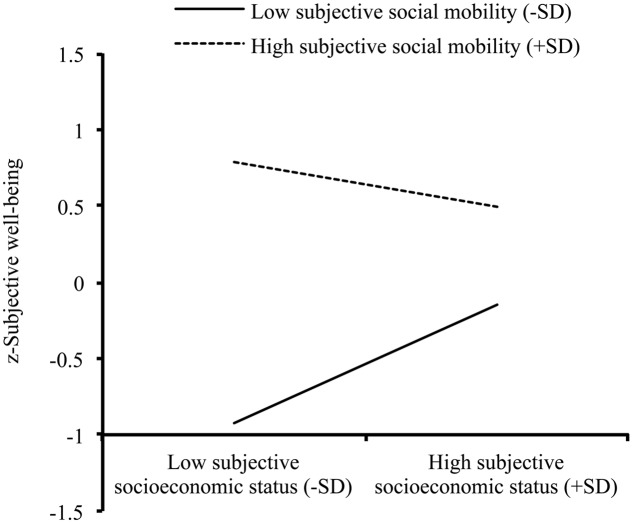
**Subjective social mobility as a moderator of the association between subjective socioeconomic status and subjective well-being**.

The mediation model tested in **Figure 1A** was extended by adding subjective social mobility as a moderator. As displayed by **Figure 1B**, the moderated mediation model (*R^2^* = 0.17, *p* < 0.001) showed that the interaction between subjective social mobility and objective SES (*c*_3_′) and the interaction between subjective social mobility and subjective SES (*b*_2_) were both significant. For the former interaction (*c*_3_′), in supporting H3a, objective SES was positively associated with subjective well-being in the high subjective social mobility condition, β = 0.45, *t* = 3.451, *p* = 0.001, but not in the low subjective social mobility condition, β = 0.11, *t* = 0.844, *p* = 0.399, as displayed in **Figure 2**. For the latter interaction (*b*_2_), in supporting H3c, subjective SES was positively correlated with subjective well-being in the low subjective social mobility condition, β = 0.39, *t* = 3.083, *p* = 0.002, but not in the high subjective social mobility condition, β = -0.15, *t* = -0.186, *p* = 0.236 (see **Figure 3**). Correspondingly, the conditional indirect effect of objective SES on subjective well-being through subjective SES in the low subjective social mobility condition was also significant, γ = 0.06, *SE* = 0.03, 95%CI [0.01, 0.15], but not in the high subjective social mobility condition, γ = -0.02, *SE* = 0.03, 95%CI [-0.08, 0.01]. Finally, the product term (objective SES × subjective social mobility, *a*_3_) was non-significant for subjective SES, which did not support H3b, indicating that the relationship between objective and subjective SES was independent of subjective social mobility.

## Discussion

The present study examined the associations between objective SES, subjective SES and subjective well-being and the moderating role of subjective social mobility among Chinese rural-to-urban migrants. In accordance with previous findings, the direct effect of objective SES on subjective well-being was significant. Additionally, in line with our hypothesis, the mediating role of subjective SES on the correlation between objective SES and subjective well-being was significant. Migrants with higher levels of objective SES might also experience higher levels of subjective SES, further providing an increase in their subjective well-being. Furthermore, we found that subjective social mobility moderates both the direct path from objective SES to subjective well-being and the indirect path from subjective SES to subjective well-being. Specifically, the direct and positive effect of objective SES on subjective well-being is only salient for migrants with higher social mobility belief. However, the indirect effect of objective SES on subjective well-being through subjective SES is only significant for the lower subjective social mobility group.

The present results confirm that attaining higher objective SES could effectively improve Chinese rural-to-urban migrants’ subjective well-being, consistent with previous investigations (Nettle, 2005; Lin, 2007; Zhang, 2007; Knight and Gunatilaka, 2010). There are two possible reasons for this result. First, Chinese rural-to-urban migrants’ objective SES improves significantly by moving from rural to urban areas, but they still stand on the bottom of the social ladder. Therefore, financial achievement would still directly raise migrants’ subjective well-being. This conclusion is supported by previous literature (Howell and Howell, 2008; Knight and Gunatilaka, 2010). Second, income is a valid and informative measure of objective SES for migrants because it not only affords the most direct assessment of migrants’ approach to valued material goods and services but also reflects the significant changes in evaluating consequences of migration (Li, 2007; Li and Li, 2010; Kraus and Stephens, 2012). Therefore, the present findings provide support for the Chinese government’s recent efforts to support new-type urbanization by showing that economic growth can directly elevate people’s subjective well-being.

In addition, our findings further uncovered a mediating role of subjective SES in the association between objective SES and subjective well-being. While most previous studies focused on the material consequences of migration, especially on income and employment (Li, 2007; Lu, 2012; China National Bureau of Statistics [CNBS], 2015), they neglected the role of subjective SES. Our findings provide evidence for the first time that subjective SES may be an important predictor of subjective well-being among Chinese rural-to-urban migrants, as well as subjective and physiological health (Demakakos et al., 2008; Gong et al., 2012; Quon and McGrath, 2014). Kraus and Stephens (2012) reported that SES is more than simply how much one has; what is more important is how much one believes one has. Although previous studies found that migrants’ subjective well-being decreased with higher expectations in relation to actual lower achievement (Knight and Gunatilaka, 2010), or by perceived discrimination (Wang et al., 2010; Chen, 2013), few investigations directly explored this hypothesis. The present findings extend this research and differentiate the effects of objective SES and subjective SES on Chinese rural-to-urban migrants’ subjective well-being. Specifically, our findings show that in addition to the direct impact of objective SES on subjective well-being, there is another indirect route via subjective SES’ mediation. Together, our findings shed light on the complicated picture of the impact of one’s social status on subjective well-being, supporting future intervention programs by recommending that both migrants’ objective and subjective SES should be considered in social welfare programs.

However, although the correlation between objective SES and subjective SES remained significant even after the Benjamini–Hochberg procedure was applied to control for the Type I error accumulation, the coefficient was very low. There are three possible reasons for this result. First, objective SES and subjective SES may represent different aspects of one’s SES. The former measures how much one actually possesses, while the latter is how much one believes he or she possesses relative to others (Boyce et al., 2010; Kraus and Stephens, 2012). The relative standard selected to make the comparison is crucial and may cause mixed results. Second, previous research has suggested that the association between objective and subjective SES differs among ethnic groups (Ostrove et al., 2000). It is possible that the weak correlation may reflect a large difference between Chinese rural-to-urban migrants and normal residents, raising a question for future investigations. Finally, only income was used when measuring objective SES in the present study. Although previous literature revealed only a moderate correlation between the objective and subjective statuses (Adler et al., 2000; Ostrove et al., 2000; Gong et al., 2012), it may still be possible that the correlation would be stronger if all three measures of objective SES were used (Adler et al., 2000).

In addition, the mediating effect of subjective SES in the present study is much weaker than the direct effect of objective SES. One possible reason is the presence of other mediating factors. For example, recent studies consistently found that compared with SES, sociometric status (defined as the level of respect and admiration individuals attain in society) had a significant impact on subjective well-being (Boyce et al., 2010; Anderson et al., 2012). Another possible reason is that some setbacks may undermine this indirect path, such as experience of discrimination and over-expectation about migration (Knight and Gunatilaka, 2010; Chen, 2013). Therefore, future investigations should continue to explore the mediating effects of these factors.

To date, no research has examined the effects of subjective social mobility on subjective well-being among Chinese rural-to-urban migrants. However, the present finding is in line with previous studies documenting the positive effect of subjective social mobility on subjective well-being among residential participants (Fischer, 2009; Nikolaev and Burns, 2014). After all, upward social mobility is a powerful motivation that drives millions of migrants to move to towns and cities. Migration theory also posits that migrating is a strategy of social mobility in itself (Knight and Gunatilaka, 2010; Chen, 2013; Jin, 2016). Our results add to this theoretic framework.

It is intriguing that subjective social mobility exhibited differential moderating effects on the above direct and indirect paths. Compared with migrants reporting lower levels of subjective social mobility, the effect of objective SES on subjective well-being only displayed significance among those reporting higher levels of subjective social mobility. However, the significant effect of subjective SES on subjective well-being was only present for migrants reporting lower levels of subjective social mobility. These findings provide an effective explanation for the controversy about the relationship between SES and subjective well-being among migrants. It is possible that someone holding a stronger sense of social mobility may concentrate more on observable achievements that could evidence their “success.” In contrast, someone with weaker subjective social mobility may rely on more subjective feelings. Either way, migrants can appreciate an elevated sense of subjective well-being. We believe there may be more factors such as subjective social mobility awaiting future investigations that add to the complexity of the impact of SES on subjective well-being.

### Limitations and Future Directions

Some caveats must be addressed in interpreting the present results. First, we analyzed our results using a set of cross-sectional data; thus, the causality in the associations found in this study is uncertain. The results suggest remarkable tendencies, but causal relationships between these variables should be confirmed by replications and be examined by longitudinal and experimental (intervention) research in the future. Second, as shown above, only using income to represent objective SES may cause a low correlation between objective and subjective SES. The three indices of SES—educational attainment, occupation, and household income—should all be included to avoid conflicts in future research. Third, although we distinguished subjective SES from objective SES, these measures still focused on the material form of social status. As suggested by other researchers, sociometric status is an alternative dimension of social status, reflecting the levels of respect and appreciation one achieves in society. Previous investigations have found that sociometric status has a stronger effect on subjective well-being than does SES (Boyce et al., 2010; Anderson et al., 2012). Therefore, future research might extend the effects of SES and explore the effect of sociometric status. Fourth, in the present study, we only focused on the positive effects of SES and subjective social mobility on migrants’ subjective well-being, neglecting multiple negative factors that may damage migrants’ subjective well-being, such as perceived discrimination. More valid models including positive and negative factors should be developed in future studies. Finally, one must be cautious in generalizing our findings because the sample in this study came from Chinese rural-to-urban migrants in Beijing, a super-city in a developing country. Further evidence needs to be presented from other diverse types of cities.

## Conclusion

The present study confirmed the significant and positive association between objective SES and subjective well-being and further showed that subjective SES partially mediated this relationship among Chinese rural-to-urban migrants. Additionally, the moderating roles of subjective social mobility were present both on the direct path from objective SES to subjective well-being and the indirect path from subjective SES to subjective well-being.

## Ethics Statement

This study was carried out in accordance with the recommendations of Beijing Normal University committee with written informed consent from all subjects. All subjects gave written informed consent in accordance with the Declaration of Helsinki. The protocol was approved by the Beijing Normal University committee.

## Author Contributions

SH, JH, and HZ conceived and designed the study. SH, JH, LS, HZ, and DD performed the investigation. SH executed the statistical analyses. SH completed the manuscript. HZ, SH, and XL modified the manuscript.

## Conflict of Interest Statement

The authors declare that the research was conducted in the absence of any commercial or financial relationships that could be construed as a potential conflict of interest.

## Footnotes

1 http://www.afhayes.com

## References

[B1] Adler N. E. Epel E. S. Castellazzo G. Ickovics J. R. (2000). Relationship of subjective and objective social status with psychological and physiological functioning: preliminary data in healthy white women. *Health Psychol.* 19 586–592. 10.1037/0278-6133.19.6.586 11129362

[B2] Anderson C. Kraus M. W. Galinsky A. D. Keltner D. (2012). The local-ladder effect social status and subjective well-being. *Psychol. Sci.* 23 764–771. 10.1177/0956797611434537 22653798

[B3] Beijing Municipal Bureau of Statistics [BMBS] (2015). *Population Census Data of Beijing in 2015.* Available at: http://www.bjstats.gov.cn/nj/main/2015-tjnj/zk/indexch.htm

[B4] Benjamini Y. Hochberg Y. (1995). Controlling the false discovery rate: a practical and powerful approach to multiple testing. *J. R. Stat. Soc. Ser. B Methodol.* 57 289–300.

[B5] Boyce C. J. Brown G. D. Moore S. C. (2010). Money and happiness rank of income, not income, affects life satisfaction. *Psychol. Sci.* 21 471–475. 10.1177/0956797610362671 20424085

[B6] Chen J. (2013). Perceived discrimination and subjective well-being among rural-to-urban migrants in China. *J. Sociol. Soc. Welf.* 40 131–156.

[B7] China National Bureau of Statistics [CNBS] (2015). *National Investigation Bulletin of Migrants in 2014.* Available at : http://www.stats.gov.cn/tjsj/zxfb/201504/t20150429_797821.html

[B8] Clark A. E. Oswald A. J. (1994). Unhappiness and unemployment. *Econ. J.* 104 648–659. 10.2307/2234639

[B9] Curhan K. B. Levine C. S. Markus H. R. Kitayama S. Park J. Karasawa M. (2014). Subjective and objective hierarchies and their relations to psychological well-being: a U.S./Japan comparison. *Soc. Psychol. Pers. Sci.* 5 855–864. 10.1177/1948550614538461 PMC4266948 25530829

[B10] Demakakos P. Nazroo J. Breeze E. Marmot M. (2008). Socioeconomic status and health: the role of subjective social status. *Soc. Sci. Med.* 67 330–340. 10.1016/j.socscimed.2008.03.038 18440111 PMC2547480

[B11] Den Berg M. V. (2011). Subjective social mobility: definitions and expectations of ‘moving up’ of poor Moroccan women in the Netherlands. *Int. Sociol.* 26 503–523. 10.1177/0268580910393042

[B12] Diener E. Lucas R. E. Oishi S. (2002). “Subjective well-being: the science of happiness and life satisfaction,” in *Handbook of Positive Psychology*, eds Snyder C. R. Lopez S. J. (New York, NY: Oxford University Press), 463–473.

[B13] Diener E. Suh E. M. Lucas R. E. Smith H. L. (1999). Subjective well-being: three decades of progress. *Psychol. Bull.* 125 276–302. 10.1037/0033-2909.125.2.276

[B14] Du Z. Liu L. L. (2011). Relationship between social support, personality and subjective well-being in migrant workers. *China J. Public Health* 27 1302–1304.

[B15] Easterlin R. A. Morgan R. Switek M. Wang F. (2012). China’s life satisfaction, 1990–2010. *Proc. Natl. Acad. Sci. U.S.A.* 109 9775–9780. 10.1073/pnas.1205672109 22586096 PMC3382525

[B16] Fischer J. A. V. (2009). *The Welfare Effects of Social Mobility: An Analysis for OECD Counties.* Available at: https://www.researchgate.net/publication/46446966_The_Welfare_Effects_of_Social_Mobility_An_Analysis_for_OECD_countries?enrichId=rgreq-0f61cac1422593e9c810ac37be95a637-XXX&enrichSource=Y292ZXJQYWdlOzQ2NDQ2OTY2O0FTOjEwMzkxNzk4NzgyNzcxNEAxNDAxNzg3Mzg4MTM1&el=1_x_3&_esc=publicationCoverPdf

[B17] Gong F. Xu J. Takeuchi D. T. (2012). Beyond conventional socioeconomic status: examining subjective and objective social status with self-reported health among Asian immigrants. *J. Behav. Med.* 35 407–419. 10.1007/s10865-011-9367-z 21720827

[B18] Hayes A. F. (2013). *An Introduction to Mediation, Moderation, and Conditional Process Analysis: A Regression- Based Approach.* New York, NY: Guilford Press.

[B19] Hayes A. F. (2016). *Process Version 2.16.2.* Available at: http://www.processmacro.org/index.html

[B20] Howell R. T. Howell C. J. (2008). The relation of economic status to subjective well-being in developing countries: a meta-analysis. *Psychol. Bull.* 134 536–560. 10.1037/0033-2909.134.4.536 18605819

[B21] Huang S. L. Han M. Sun L. Shang R. (2016). The effects of belief in a just world on the sense of social responsibility of college students: the mediating role of the belief of social mobility. *J. Beijing Norm. Univ. Soc. Sci.* 68–74.

[B22] Jin L. (2016). Migration, relative deprivation, and psychological well-being in China. *Am. Behav. Sci.* 60 750–770. 10.1177/0002764216632826

[B23] Johnson S. E. Richeson J. A. Finkel E. J. (2011). Middle class and marginal? Socioeconomic status, stigma, and self-regulation at an Elite university. *J. Pers. Soc. Psychol.* 100 838–852. 10.1037/a0021956 21280968

[B24] Kelley S. Kelley C. (2009). *Subjective Social Mobility: Data from 30 Nations. Charting the Globe: The International Social Survey Programme 2009.* Available at: http://internationalsurvey.org/Subjective%20Social%20Mobility-Data%20from%2030%20Nations.pdf

[B25] Knight J. Gunatilaka R. (2010). Great expectations? The subjective well-being of rural-urban migrants in China. *World Dev.* 38 113–124. 10.1016/j.worlddev.2009.03.002

[B26] Kraus M. W. Adler N. Chen T.-W. D. (2013a). Is the association of subjective SES and self-rated health confounded by negative mood? An experimental approach. *Health Psychol.* 32 138–145. 10.1037/a0027343 22329426 PMC3565057

[B27] Kraus M. W. Côté S. Keltner D. (2010). Social class, contextualism, and empathic accuracy. *Psychol. Sci.* 21 1716–1723. 10.1177/0956797610387613 20974714

[B28] Kraus M. W. Piff P. K. Keltner D. (2011). Social class as culture the convergence of resources and rank in the social realm. *Curr. Dir. Psychol. Sci.* 20 246–250. 10.1177/0963721411414654

[B29] Kraus M. W. Stephens N. M. (2012). A road map for an emerging psychology of social class. *Soc. Pers. Psychol. Compass* 6 642–656. 10.1111/j.1751-9004.2012.00453.x

[B30] Kraus M. W. Tan J. J. Tannenbaum M. B. (2013b). Locating the social ladder across cultures and identities. *Psychol. Inq.* 24 131–134. 10.1080/1047840X.2013.799989

[B31] Lei J. Tam T. (2012). *Subjective Social Status, Perceived Social Mobility and Health in China.* Available at: http://paa2012.princeton.edu/papers/122792

[B32] Li C. (2007). Socioeconomic status attainment of rutal-urban migration. *J. Beijing Univ. Technol.* 7 5–10.

[B33] Li P. L. Li W. (2010). The economic status and social attitudes of migrants in recent years. *Soc. Sci. China* 119–131.

[B34] Lin X. (2007). Investigation of subjective well-being among Chinese migrants. *J. Nanjing Coll. Popul. Programme Manag.* 23 35–37. 10.5588/ijtld.13.0866

[B35] Lu F. (2012). Wage trends among Chinese migrant workers: 1979-2010. *Soc. Sci. China* 7 47–67.

[B36] McGillivray M. (2007). “Human well-being: issues, concepts and measures,” in *Human Well-Being: Concept and Measurement*, ed. McGillivray M. (London: Palgrave Macmillan), 1–22.

[B37] Ming L. (2013). *The Relationship of Characteristics of Both Institutional Trust and Social Mobility Beliefs with Student Engagement.* Master’s dissertation, Central University of Finance and Economics, Beijing.

[B38] Nettle D. (2005). *Socio-economic Status and Subjective Well-Being.* Newcastle upon Tyne: New Castle University.

[B39] Nikolaev B. Burns A. (2014). Intergenerational mobility and subjective well-being evidence from the general social survey. *J. Behav. Exp. Econ.* 53 82–96. 10.1016/j.socec.2014.08.005

[B40] Ostrove J. M. Adler N. E. Kuppermann M. Washington A. E. (2000). Objective and subjective assessments of socioeconomic status and their relationship to self-rated health in an ethnically diverse sample of pregnant women. *Health Psychol.* 19 613–618. 10.1037/0278-6133.19.6.613 11129365

[B41] Quon E. C. McGrath J. J. (2014). Subjective socioeconomic status and adolescent health: a meta-analysis. *Health Psychol.* 33 433–447. 10.1037/a0033716 24245837 PMC5756083

[B42] Ramzy A. (2009). *The Chinese Worker.* Available at: http://content.time.com/time/specials/packages/article/0,28804,1946375_1947252_1947256,00.html

[B43] Van Hoorn A. A. J. Oecd. (2008). “A short introduction to subjective well-being: measurement, correlates and policy uses,” in *Statistics, Knowledge and Policy 2007: Measuring and Fostering the Progress of societies*, ed. OECD (Paris: OECD Publishing), 215–229.

[B44] Wang B. Li X. Stanton B. Fang X. (2010). The influence of social stigma and discriminatory experience on psychological distress and quality of life among rural-to-urban migrants in China. *Soc. Sci. Med.* 71 84–92. 10.1016/j.socscimed.2010.03.021 20403653

[B45] Watson D. Clark L. A. Tellegen A. (1988). Development and validation of brief measures of positive and negative affect: the PANAS scales. *J. Pers. Soc. Psychol.* 54 1063–1070. 10.1037/0022-3514.54.6.1063 3397865

[B46] Xing Q. Ma Y. Kong L. Zi F. (2012). Relationship between self-esteem and subjective well-being of peasant workers. *China J. Health Psychol.* 20 1809–1811.

[B47] Zhang X. (2007). Analyzing peasant-workers’ happiness sense: take a example of 521 peasant-workers who in wuhan city. *J. Soc. Work* 48–51.

[B48] Zhu J. (2010). The marginalization of the Chinese peasants and peasant-workers: an empirical data analysis based on the number and income of peasant-workers. *J. Northwest A F Univ. Soc. Sci. Edn.* 10 9–14.

